# Antioxidant Electrospun Poly(3-hydroxybutyrate-*co*-3-hydroxyvalerate) (PHBV) Films Loaded with Resveratrol Solubilized in Deep Eutectic Solvents

**DOI:** 10.1021/acsapm.5c01101

**Published:** 2025-08-07

**Authors:** Ahmet O. Basar, Cristina Prieto, Evangelia Bardakou, Luis Cabedo, Jose M. Lagaron

**Affiliations:** † Novel Materials and Nanotechnology Group, Institute of Agrochemistry and Food Technology (IATA), Spanish Council for Scientific Research (CSIC), Calle Catedrático Agustín Escardino Benlloch 7, Paterna 46980, Spain; ‡ Polymers and Advanced Materials Group (PIMA), School of Technology and Experimental Sciences, Universitat Jaume I (UJI), Avenida de Vicent Sos Baynat s/n, Castellón 12071, Spain

**Keywords:** PHBV, DES, resveratrol, electrospinning, antioxidants, active packaging

## Abstract

The present study explores the remarkable capabilities of deep eutectic solvents (DES) for enhancing the antioxidant properties of films containing the natural antioxidant resveratrol (Res). A key achievement of this work is the unprecedented solubility of resveratrol—400 mg/mL—in a highly effective DES composed of choline chloride (ChCl) and ethylene glycol (EG), representing a significant enhancement over previously reported values. For the active and sustainable packaging development purposes, this was coupled with the creation of continuous and nonporous electrospun poly­(3-hydroxybutyrate-*co*-3-hydroxyvalerate) (PHBV) biopaper material through electrospinning and subsequent thermal annealing processes, featuring enhanced antioxidant properties. Remarkably, a second achievement of this paper is that electrospun biopapers containing DES-solubilized Res exhibited a 30% improvement in antioxidant activity and material efficiency compared to those containing the same amount of nonsolubilized resveratrol within the polymer. Additionally, characterization was made via wide-angle X-ray diffraction, optical properties, mechanical and barrier testing, demonstrating that the studied DES not only optimized the functional attributes of PHBV biopapers but also maintained their structural integrity and mechanical and barrier performance. Therefore, this study highlights the potential of DES as a potent tool for improving the effectiveness of poorly soluble natural antioxidants, paving the way for the development of innovative solutions in active and sustainable packaging.

## Graphical abstract

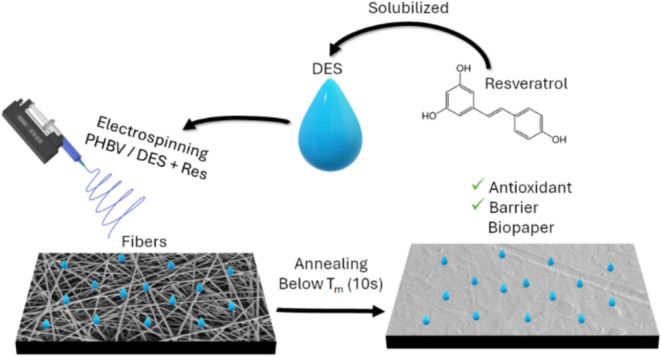

## 1 Introduction

Active packaging is a recent strategy applied in the food packaging sector to extend the shelf life or maintain and improve the condition of packaged food according to the definition of the European Commission (EC) Regulation No 450/2009,^1^ which sets out the legal framework for packaging materials that intentionally incorporate active substances to enhance food quality and safety. Among the main culprits behind food deterioration, oxidative processes lead to changes in color and texture, development of off-flavor, and a decrease in both nutritional value and overall quality of food products.^2^ In this sense, antioxidant agents tackle these issues by releasing compounds that inhibit oxidation, preserving flavor, safety, and nutritional integrity.^3^ Incorporating natural antioxidants into biodegradable polymeric films represents an interesting approach to improve the functionality and usefulness of food packaging materials, with a focus on environmental sustainability. Over the past decade, the food industry has utilized various synthetic antioxidants, such as butylated hydroxyanisole (BHA), butylated hydroxytoluene (BHT), propyl gallate, and *tert*-butyl hydroquinone.^4^ However, increasing health concerns and consumer demands for natural alternatives have prompted researchers to seek safer, natural options.^5,6^

Resveratrol (trans-3,4,5-trihydroxystilbene) is a naturally occurring polyphenol found in several plant species, including grapes, mulberries, and peanuts, among others.^7^ This molecule stands out due to its significant health benefits and protective effects against oxidative stress. The antioxidant capacity of resveratrol is of particular interest in food packaging industry given its ability to effectively scavenge free radicals, including superoxide radicals (O_2_^–^), hydroxyl radicals (OH^–^), hydrogen peroxide (H_2_O_2_), nitric oxide (NO), and nitrogen dioxide (NO_2_).^8^ Besides its excellent antioxidant capacity, resveratrol has been also proven to possess antimicrobial properties, which is relevant to food packaging applications.^9^ However, the application of resveratrol—as well as many other natural active compounds—in food industry presents challenges, particularly due to its thermal instability and difficulty in achieving homogeneous distribution within polymer matrices.^10^ To address the latter issue, one potential strategy is to solubilize resveratrol in a suitable solvent. Resveratrol is soluble in alcohols and various organic solvents, such as ethanol (50 mg/mL), dimethylformamide (65 mg/mL), and dimethyl sulfoxide (16 mg/mL), but its solubility in aqueous or lipid phases is limited, with trace amounts dissolving in water (0.023 mg/mL) and coconut oil (0.18 mg/mL).^10–12^ Given the low to moderate solubility of resveratrol, adopting innovative, cost-effective, environmentally friendly, non- or low-toxic, and renewable deep eutectic solvents (DES) offers a promising alternative. DESs are defined as eutectic mixtures of hydrogen bond acceptor (HBA) and hydrogen bond donor (HBD), which form a homogeneous liquid that remains liquid at temperatures significantly below the melting points of its individual constituents and their ideal mixtures.^13^ These solvents have proven to be valuable in a wide array of fields as eco-friendly and sustainable alternatives to conventional organic solvents and ionic liquids.^14^ Their advantageous characteristics—depending on the precursors used—include the use of low-cost precursors, biodegradability, biocompatibility, straightforward production processes, minimal or no toxicity.^13,14^ Additionally, their physicochemical characteristics, such as viscosity, polarity, and hydrogen bonding capacity, can be finely tuned, making DESs highly versatile media for solubilizing a broad spectrum of compounds with high capacity and enhanced stabilization.^13,15^

In alignment with the sustainability values offered by deep eutectic solvents (DES), it is equally critical to prioritize the use of sustainable polymeric materials in the design of active food packaging materials. In this context, poly­(3-hydroxybutyrate-*co*-3-hydroxyvalerate) (PHBV), belonging to the polyhydroxyalkanoates (PHAs) family, emerges as a notable contender. This compound is a naturally occurring, biobased, biodegradable, and biocompatible aliphatic polyester, synthesized by microorganisms and comprising the homopolymer poly­(3-hydroxybutyrate) (PHB) augmented with hydroxyvalerate (HV) units along its structural backbone.^15,16^ As one of the foremost alternatives to fossil fuel-derived polymers, PHBV exhibits medium oxygen and high-water vapor barrier properties, positioning it as a significant candidate for novel packaging solutions that are designed to mitigate the escalating global concerns over environmental health.^17,18^ However, the primary challenge associated with the use of PHAs in the food industry lies in their vulnerability to high temperatures during the manufacturing process, as their thermal degradation occurs close to their melting point.^19^

In polymer-based food packaging, as well as in the specific case of PHAs, the most commonly employed processing techniques are extrusion, injection molding, and thermoforming.^1^ These processes involve the melting of the polymeric material and subsequent shaping into a continuous product. However, these methods also require the use of elevated temperatures over extended periods. As previously mentioned, this poses a significant challenge for active compounds, particularly those derived from natural sources, as exposure to high temperatures can result in their degradation, thereby diminishing their bioactivity,^1,20^ which similarly affects PHAs. In addition to these, these conventional processes possess other disadvantages, including production of thick material layers, instability of active compounds during processing, and challenges in scaling up.^20,21^ Against this backdrop, electrospinning process emerges as an innovative approach for fabricating polymeric materials, garnering particular interest in the food packaging industry. Electrospinning is a straightforward and flexible method that achieves the vaporization of organic solvents at room temperature from a polymeric solution through the application of a high electrical potential. This process results in the creation of ultrathin, fibrous polymeric materials characterized by a high surface-to-volume ratio and adjustable pore sizes.^15^ Moreover, because electrospinning occurs at ambient temperatures, it allows for the inclusion of thermosensitive substances, such as resveratrol, ensuring their stability and homogeneous distribution within the polymer matrix.^22^ In the specific context of packaging applications, the electrospun fibers can undergo a postprocessing thermal step below the polymer melting point to control interfiber coalescence, hence porosity, to a level such that the nanofibers evolved into a continuous film, so-called biopapers,^23,24^ which exhibit high barrier properties. This process involves a short-duration exposure (typically 5–10 s) to temperatures below the polymer’s melting point which minimize any potential thermal degradation of thermosensitive components like PHAs and resveratrol.^17,18,22,25,26^

In this study, we focused on solubilization of resveratrol in deep eutectic solvents for the development of fully eco-friendly continuous electrospun PHBV biopapers with antioxidant properties for active food packaging applications. Additionally, the developed biopapers were evaluated to understand their structural, thermal, and mechanical attributes, alongside their crystallinity, barrier capabilities, and antioxidant activities, all of which hold significant relevance to their application in food packaging.

## 2 Experimental Section

### 2.1 Materials

The natural antioxidant compound, resveratrol 98% (HPLC grade), extracted from Giant Knotweed, was purchased from JIAHERB (Xi’an, China). Commercial poly­(3-hydroxybutyrate-*co*-3-hydroxyvalerate) (PHBV), ENMAT Y1000P, was purchased from Tianan Biologic Materials (Ningbo, China). The polymer resin was in the form of pellets with a density, molecular weight (Mw), and 3HV fraction of 1.23 g/cm^3^, ∼ 2.8 × 10^5^ g/mol, and 2–3 mol %, respectively. 2,2,2-trifluorethanol (TFE) (≥99%) was purchased from Merck (Darmstadt, Germany). Choline chloride (ChCl) (≥98%), urea (≥99%), citric acid (CA) (99%), ethylene glycol (EG) (99.8%), poly­(ethylene glycol) 200 (PEG), and 2,2-Diphenyl-1-picrylhydrazyl radical (DPPH) were purchased from Sigma-Aldrich (St. Louis, MO). Glycerol (Gly) (pure, pharma grade) was purchased from AppliChem GmbH (Darmstadt, Germany). Finally, methanol HPLC grade were obtained from Panreac (Barcelona, Spain). Distilled water was used throughout the study.

### 2.2 Preparation of Deep Eutectic Solvents

Deep eutectic solvents (DES) were prepared by mixing the individual components, namely the hydrogen bond donor (HBD) and the hydrogen bond acceptor (HBA), in accordance with their respective molar ratios. Prior to mixing, choline chloride (ChCl) was dried at 65 °C under vacuum (Vaciotem-T, JP Selecta, Barcelona, Spain) for 24 h to remove any possible moisture. Five different DESs, namely ChCl/Urea/Water, ChCl/Gly, ChCl/EG, EG/CA, PEG/CA, were synthesized by weighting the individual components in appropriate mole ratios of 1:2:1, 1:2, 1:2, 4:1, and 4:1, respectively. The mixtures were then heated at 80 °C under constant stirring until a clear and colorless liquid was obtained. The resulting DESs retained their liquid state even after cooling to room temperature for several months and were stored in desiccator until further use.

### 2.3 Solution Preparation

Solubility of resveratrol was studied visually by adding it into prepared DESs with gentle stirring at room temperature. Resveratrol was continuously added until turbidity was observed. Next, electrospinning solution was prepared by dissolving 10 wt % of PHBV in TFE at 50 °C through gentle stirring. Thereafter, a correct amount of DES-solubilized resveratrol was added into PHBV solution to attain a final Res-to-PHBV ratio of 1 wt %. As controls, solid resveratrol (1 wt % in PHBV) and pure DES were directly added into the electrospinning solution. For the latter, the amount of DES-to-PHBV was adjusted to match the proportion of Resveratrol-solubilized DES that provides 1 wt % of resveratrol, that was 2.6 wt % DES in PHBV. All blend solutions were homogenized using a TX4 Digital Vortex Mixer from Velp (Usmate, Italy) for 3 min.

### 2.4 Electrospinning Process

Electrospinning process was conducted using a high-throughput Fluidnatek LE-500 pilot tool, an electrospinning apparatus from Bioinicia S.L. (Valencia, Spain), configured for the lab mode with a single needle injection. Each solution was introduced into the electrospinning equipment using a 20 mL plastic syringe connected to a stainless-steel needle with inner diameter of 0.4 mm, which was in turn connected to the power supply. For the electrospinning of each solution, the distance and flow rate were optimal at 15 cm, and 6 mL/h, respectively. The applied voltage for the electrospinning process was 12 kV for pure PHBV and resveratrol-containing PHBV solution, while it was slightly higher at 16 kV for the DES-containing PHBV solution. All electrospinning processes were performed at 50% relative humidity (RH) and 25 °C conditions. The resultant fiber mats were kept in a desiccator conditioned at 0% RH and room temperature at least 2 weeks prior to the annealing process.

### 2.5 Preparation of Biopapers—Annealing

The resultant electrospun mats were subjected to a thermal post-treatment, so-called annealing, below the biopolymer’s melting temperature using a 4122-model press from Carver, Inc. (Wabash, IN). Based on previous studies, the annealing parameters were optimized at 155 °C for 10 s without applying pressure.^26^ The average thickness for all electrospun biopapers was approximately 80 μm. Before undergoing further characterization, all produced biopapers were carefully stored in a desiccator at 0% RH for a minimum of 2 weeks.

### 2.6 Characterizations

#### 2.6.1 Morphology and EDX Analysis

The morphologies of the top view and cross sections of the electrospun biopapers were examined by field emission scanning electron microscopy (FESEM) using a FEI SCIOS 2 Dual Beam electron microscope (Thermo Fischer Scientific, Waltham, MA) at an accelerating voltage of 3 kV and working distance of 6.9 mm. For this, the samples were previously placed onto holders using conductive double-sided adhesive tape and sputtered with a gold–palladium mixture for 2 min under vacuum. For the cross-section imaging, the samples were cryo-fractured after being fully frozen in liquid nitrogen. The average fiber diameter was measured using the ImageJ Launcher software program (NIH) (Bethesda, MD) from the obtained SEM images in their original magnifications.

The chemical composition was analyzed and visualized using an energy dispersive X-ray (EDX) detector (Oxford Instruments, Oxfordshire, U.K.) connected to the Philips ESEM XL30 electron microscope (Amsterdam, Netherlands) with an accelerating voltage of 20 kV. Elemental mapping specifically focused on the element chlorine (Cl).

#### 2.6.2 Thermal Analysis

Thermogravimetric analysis (TGA) was performed for all electrospun biopaper samples in a nitrogen atmosphere using a 550-TA Instruments thermogravimetric analyzer (New Castle, DE). The protocol involved heating from 25 to 700 °C at a rate of 10 °C/min. All tests were performed in triplicate. TA TRIOS software (TA Instruments, New Castle, DE) was utilized for the analysis of all thermogravimetric data.

The thermal transitions of the examined samples were assessed using differential scanning calorimetry (DSC) with a Q20 instrument from TA Instruments (New Castle, DE) in a nitrogen atmosphere. Approximately 3 mg of each sample were placed in a Tzero hermetic aluminum pan sealed. All thermal runs were conducted at a rate of 10 °C/min and consisted of an initial heating step from −20 to 190 °C, followed by a cooling step to −20 °C, and a second heating step to 190 °C, with 60 s isothermal holds between the runs. Prior to analysis, the DSC instrument was calibrated using indium as a standard. Each measurement was carried out in triplicate, and all thermograms were analyzed using TA Universal Analysis 2000 software (TA Instruments, New Castle, DE).

#### 2.6.3 WAXD

Wide-angle X-ray diffraction (WAXD) analysis was performed at room temperature for all electrospun biopaper samples using a Bruker AXS D4 ENDEAVOR diffractometer (Billerica, MA). The biopapers were investigated using reflection mode, utilizing incident Cu K-alpha radiation with a wavelength (*k*) of 1.54 Å. Generator settings were adjusted to 40 kV and 40 mA. Data collection encompassed a scattering angle (2θ) range of 2–40°.

#### 2.6.4 Transparency

The light transmission of the electrospun biopaper samples was measured using ultraviolet–visible (UV–vis) spectrophotometer VIS3000 (Dinko Instruments, Barcelona, Spain). For this, the samples were cut into the dimension of 50 × 30 mm, and their light absorption was quantified at wavelengths between 200 and 700 nm. Transparency (*T*) values were calculated using eq 1.^27^

1 $$T=10^{\left(2-A_{600}\right)}$$

where *A*_600_ corresponds to the absorbance at 600 nm.

#### 2.6.5 Tensile Tests

Mechanical parameters of the electrospun biopaper samples were characterized using an Instron 4400 universal testing machine (Norwood, MA) according to ASTM D638. The samples were prepared in a dog-bone shape with dimensions of 5 × 25 mm. Prior to analysis, the samples were conditioned to the test conditions (40% RH, 25 °C) for at least 1 day. All tensile tests were carried out using six identical specimens for each specimen.

#### 2.6.6 Permeability

The water vapor permeability (WVP) of the electrospun biopaper samples was determined using the standard, ASTM E96–95. For this, Payne permeability cups from Elcometer Sprl (Hermallesous-Argenteau, Belgium) with a diameter of 35 cm were utilized. In each cup, 5 mL of distilled water were introduced. The biopapers were securely positioned with silicon rings, ensuring no direct contact with water, and exposed to 100% RH on one side only. Subsequently, the cups were placed in a desiccator sealed with dried silica gel, conditioned at 0% RH and 25 °C. Periodic weight measurements were taken using an analytical balance (±0.0001 g). The WVP was determined by analyzing the data on weight loss over time, where the weight loss was calculated by deducting the loss through sealing from the total loss. The permeability values were derived by multiplying the permeance by the thickness of the biopaper.

For the case of oxygen permeability (OP), its coefficient was derived by measuring the oxygen transmission rate (OTR) utilizing an Oxygen Permeation Analyzer M8001 manufactured by Systech Illinois (Thame, U.K.). Prior to testing, the samples were equilibrated to the specified humidity and purged with nitrogen. Subsequently, they were subjected to an oxygen flow rate of 10 mL/min, with a test area of 5 cm^2^ for each sample. The calculation of OP took into consideration the biopaper thickness and partial gas pressure.

#### 2.6.7 Antioxidant Activity

2,2-Diphenyl-1-picrylhydrazyl (DPPH), characterized as a stable radical, is commonly employed in the evaluation of the free radical scavenging capabilities of antioxidant compounds due to its ease of reduction by these substances. The mechanism of the assay relies on the reduction of DPPH through an antioxidant that acts as an electron or hydrogen atom donor.^28^ In this regard, resveratrol, a well-known phenolic compound, demonstrates antioxidant activity by donating a hydrogen atom from its biphenyl groups to the radical acceptor.^7,29^

DPPH radical scavenging assay was performed to evaluate the antioxidant activity of the developed electrospun biopapers. For this, a series of vials with increasing amounts of sample were mixed with the DPPH solution (100 μM in methanol). Control vials containing only the DPPH solution were also prepared. Each vial was prepared in triplicate. After adding the DPPH solution, the vials were immediately stored in the dark at room temperature. The measurements were taken after 24 h of storage, at 517 nm using a UV 4000 spectrophotometer from Dinko Instruments. The inhibition to DPPH (%) values were determined using following eq 2 ^22^

2 $$\mathrm{inhibition}\;\mathrm{DPPH}\left(\%\right)=\frac{A_{\mathrm{control}}-A_{\mathrm{sample}}}{A_{\mathrm{control}}}\times 100$$

where *A*_control_, and *A*_sample_ are the absorbance values of the DPPH solution, and the test sample, respectively.

The results were expressed as sample weight per ml of DPPH solution, also enabling the calculation of the 50% inhibition value (IC50), which indicates the essential amount of the sample needed to reduce the absorbance intensity of DPPH by 50%.^29^ The IC50 values were obtained through regression analysis, yielding a *R* ^2^ value higher than 0.97.

#### 2.6.8 Statistical Analysis

A statistical analysis was carried out using a one-way ANOVA, supplemented by Tukey’s multiple comparison test, to ascertain significant differences at a 95% confidence level. OriginPro8.5 (OriginLab Corporation, Northampton, MA) was utilized for data analysis. In every instance, a *p-*value of 0.05 or less was deemed to indicate statistical significance.

## 3 Results and Discussion

### 3.1 Resveratrol Solubility

The solubility of resveratrol in various DES-based carriers was evaluated by dissolving it to its maximum capacity, i.e., before detecting saturation by naked eye. For this purpose, several DESs were selected, including ChCl/Urea/Water, ChCl/Gly, EG/CA, ChCl/EG, PEG/CA, and individual liquid components were also tested as controls, including pure PEG and EG. Results are presented in Table 1. Resveratrol solubility showed completely different behavior depending on the formulation of DES solvents. Among DESs, the lowest solubility was observed for EG/CA (50 mg/mL), whereas the highest solubility was achieved for ChCl/EG (400 mg/mL). Interestingly, pure EG demonstrated the lowest solubility at 50 mg/mL, indicating that the formation of a DES with choline chloride enhanced resveratrol solubility. Previously, Robinson et al. conducted a study on the solubility of resveratrol in several common solvents, reporting a maximum resveratrol solubility of 374 mg/mL in PEG-400, up to our knowledge, the highest solubility found in the literature for resveratrol.^30^ In our study, a lower solubility of 300 mg/mL in PEG-200 was obtained. In the case of PEG-based DES, concretely PEG:CA, DES formation resulted in a lower resveratrol solubility (150 mg/mL) compared to pure PEG. Therefore, it can be interpreted that each DES uniquely functions as a solubilizing agent depending on its formulation. This enhanced solubility can be attributed to the unique solvation mechanisms of DES, which deviate from traditional polarity-based solubility rules. Choline chloride-based DES form extensive hydrogen-bond networks and microheterogeneous domains that can encapsulate hydrophobic molecules, effectively shielding them from the bulk polar environment. This allows even hydrophilic DES to dissolve substantial amounts of poorly water-soluble compounds like resveratrol through nonclassical interactions such as hydrogen bonding, hydrotropy, and nanostructuring.^31,32^ For instance, Uka et al. conducted a recent study on the solubility of resveratrol in various deep eutectic solvents, including ChCl- and menthol-based formulations.^33^ Their findings indicate that the ChCl:1,2-propanediol system exhibited the highest solubility of resveratrol, reaching 211 mg/mL.

**Table 1:** Maximum Solubility of Resveratrol in Different Deep Eutectics Solvents, and Some of Their Individual Components as Solvents

solvents	maximum solubility(mg/mL)
ChCl/Urea/Water	100
ChCl/Gly	150
EG/CA	50
ChCl/EG	400
EG	50
PEG/CA	150
PEG	300

Ultimately, ChCl:EG was chosen for further study due to its highest observed resveratrol solubility. Additionally, the visualization of resveratrol-solubilized ChCl/EG is depicted in Figure S1 for each resveratrol concentration. The figure illustrates that as the resveratrol concentration increases, the color of the solution transitions to yellow/brown. Remarkably, even at the highest concentration of 400 mg/mL resveratrol, the solution remained transparent even after 2 months of storage without any precipitation.

### 3.2 Morphology

Figure 1 presents the morphology of the fiber mats postelectrospinning, including top views and cross-sectional surfaces of the biopapers obtained through the annealing process, where Figure S2 reports fiber size distribution of all fiber mats. Electrospinning PHBV-based solutions led to similar morphologies, displaying smooth, continuous, bead-free fibers. However, DES-containing PHBV fibers seemed to be slightly smaller in fiber diameter even if the differences were nonsignificant, measuring 1.37 ± 0.49, 1.15 ± 0.31, 0.97 ± 0.24, 0.83 ± 0.15 μm for pure PHBV, PHBV + Res, PHBV/ChCl:EG, PHBV/ChCl:EG + Res, respectively. Among them, PHBV/ChCl:EG+Res fibers showed the smallest average diameter and the narrowest diameter distribution, suggesting the most uniform and consistent fiber morphology, as illustrated in Figure S2. In this regard, the introduction of ChCl-based DESs could increase the solution conductivity, ultimately leading to smaller fiber diameters as it was demonstrated in a previous study.^15,34^ Furthermore, when comparing the top views of the electrospun mats before and after annealing (Figure 1, column I and II), resulted in continuous electrospun biopapers without porosity as evidenced by the cross-sectional imaging in Figure 1 (column III). Thus, it can be concluded that thermal postprocessing successfully achieved a compact packing rearrangement of the electrospun fibers by minimizing their surface energy, resulting in continuous films in each case. This morphology is of importance, particularly for food packaging applications.^25^

**Figure 1:**
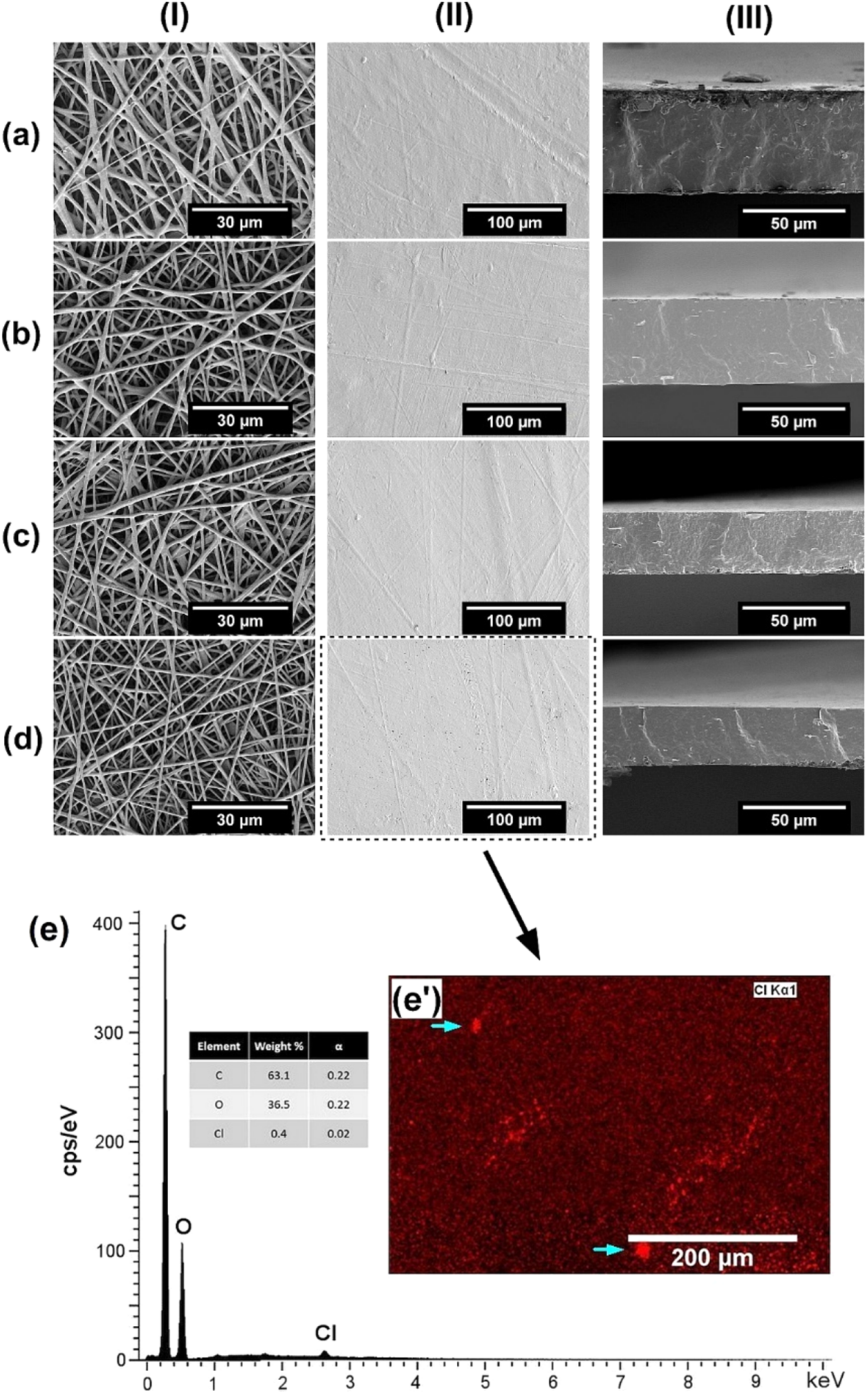
SEM images of electrospun fiber mats in the top view (I), and their biopapers in top (II) and cross-section (III) views: (a) pure PHBV, (b) PHBV + Res, (c) PHBV/ChCl:EG, (d) PHBV/ChCl:EG + Res, and EDX analysis for electrospun PHBV/ChCl:EG + Res biopaper: (e) EDX scan spectrum and (e′) elemental mapping for Cl element.

Subsequently, EDX analysis was conducted on the PHBV/ChCl:EG+Res biopaper sample to ascertain the presence and dispersion of the introduced DES, ChCl:EG, within the PHBV matrix, which can also indirectly confirm the presence of resveratrol, as it was solubilized in DES. Figure 1e displays the EDX spectrum, revealing the presence of C (63.1 wt %) and O (36.5 wt %) as major elements. In addition to that, a small quantity of Cl elements (0.4 wt %) originating from ChCl:EG was also detected. The elemental map (Figure 1e′) illustrates a relatively good homogeneous distribution of Cl elements, representing the DES and resveratrol, within the PHBV/ChCl:EG+Res biopapers. Nevertheless, higher intensity areas of DES, as indicated by the cyan arrows in Figure 1e′, might be attributed to some DES aggregation.

Additionally, complementary ATR-FTIR analysis was also performed on the developed PHBV-based biopapers, as well as on the raw materials (ChCl/EG and resveratrol); the corresponding results are presented in the Supporting Information.

### 3.3 Thermal Properties

Thermal transitions of the electrospun PHBV-based biopapers were analyzed by DSC. The thermograms of the samples during the first heating and cooling runs are displayed in Figure 2a,2b, respectively, and the corresponding data are gathered in Table 2. DSC results show that the electrospun pure PHBV biopapers showed a typical *T*_m_ and *T*_c_ of approximately 172 and 119 °C in the first heating and cooling runs, respectively, while the Δ*H*_m_ and Δ*H*_c_ values were similar to each other, averaging 105 J/g. Similar results have been reported in the literature for the electrospun pure PHBV (ENMAT Y1000P) films.^17,26^ On the other hand, pure resveratrol has a high melting point of 267 °C,^35^ which could not be detected in the DSC runs performed on PHBV-based samples due to degradation of the polymer at such elevated temperatures.^36^

**Figure 2:**
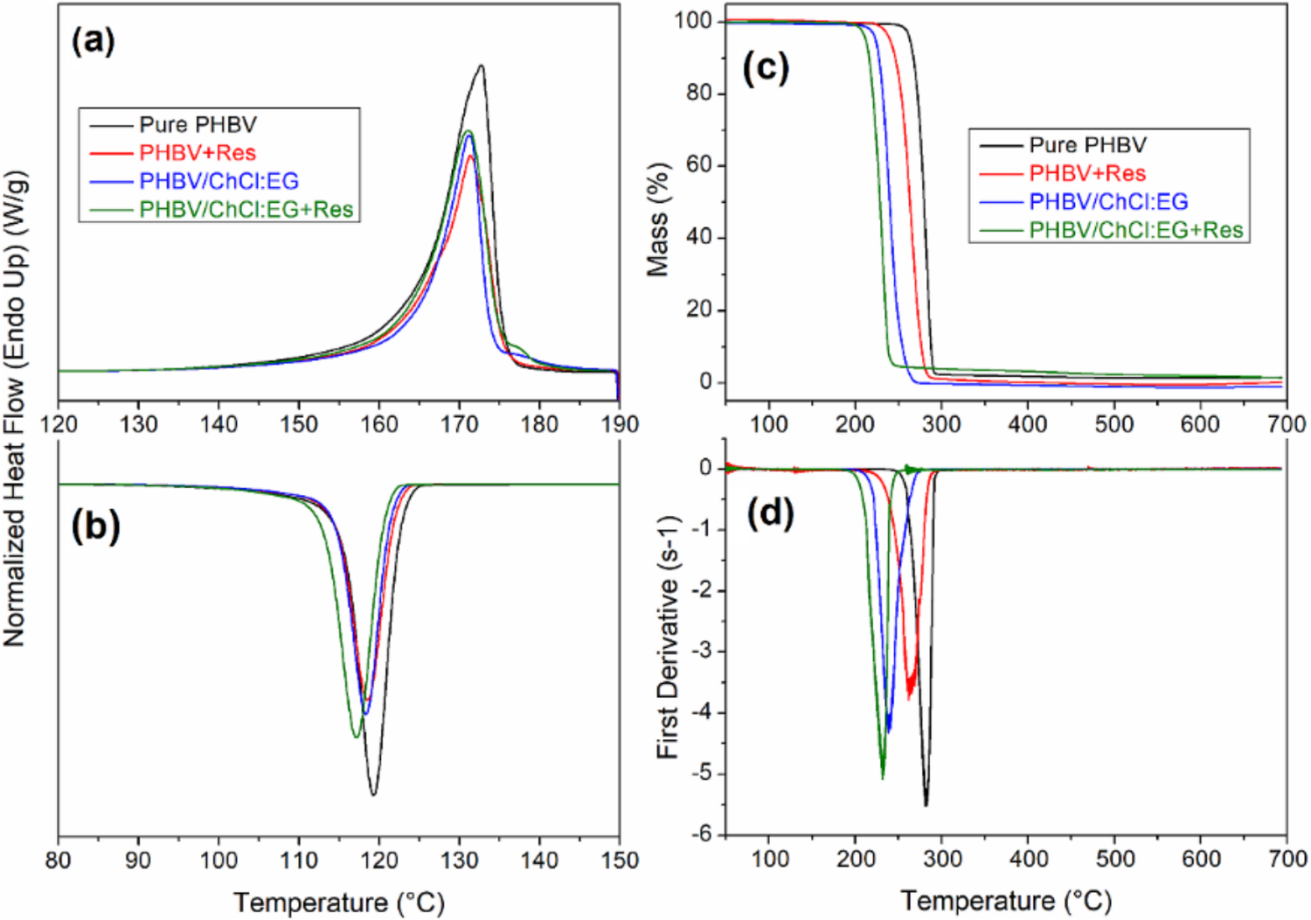
Differential scanning calorimetry (DSC) curves obtained during the (a) first heating, and (b) cooling runs of the electrospun biopaper samples: pure PHBV, PHBV + Res, PHBV/ChCl:EG, PHBV/ChCl:EG + Res. (c) Thermogravimetric analysis (TGA) and (d) first derivative (DTG) curves of the same samples.

**Table 2:** Thermal Properties of Electrospun PHBV-Based Biopapers^a^

	DSC parameters				TGA parameters			
electrospun biopaper samples	*T*_m_ (°C)	Δ*H*_m_ (J/g)	*T*_c_ (°C)	Δ*H*_c_ (J/g)	*T*_onset_ (°C)	*T*_deg_ (°C)	mass loss at *T*_deg_ (%)	residual mass at 700 °C (%)
pure PHBV	171.7 ± 1.5[Table-fn t2fn2]	104.4 ± 4.7[Table-fn t2fn2]	118.8 ± 0.7[Table-fn t2fn2]	106.6 ± 4.4[Table-fn t2fn2]	273.4 ± 4.3[Table-fn t2fn2]	285.7 ± 4.3[Table-fn t2fn2]	65.9 ± 1.0[Table-fn t2fn2]	1.2 ± 0.2[Table-fn t2fn2]
PHBV + Res	170.9 ± 0.6[Table-fn t2fn2]	79.2 ± 1.8[Table-fn t2fn2]	118.6 ± 0.1[Table-fn t2fn2]	84.1 ± 5.8[Table-fn t2fn2]	255.3 ± 10.2[Table-fn t2fn2]	269.4 ± 8.9[Table-fn t2fn2]	52.6 ± 5.9[Table-fn t2fn2]	0.9 ± 1.3[Table-fn t2fn2]
PHBV/ChCl:EG	170.8 ± 0.5[Table-fn t2fn2]	73.9 ± 4.4^c^	118.3 ± 0.1[Table-fn t2fn2]	80.4 ± 4.6[Table-fn t2fn2]	226.9 ± 1.6[Table-fn t2fn2]	238.9 ± 0.5[Table-fn t2fn2]	47.5 ± 4.6[Table-fn t2fn2]	0.2 ± 0.3[Table-fn t2fn2]
PHBV/ChCl:EG + Res	171.3 ± 0.1[Table-fn t2fn2]	78.5 ± 14.6[Table-fn t2fn2]	117.5 ± 0.5[Table-fn t2fn2]	81.4 ± 15.4[Table-fn t2fn2]	228.8 ± 6.4[Table-fn t2fn2]	235.9 ± 5.6[Table-fn t2fn2]	51.7 ± 20.0[Table-fn t2fn2]	2.4 ± 0.1[Table-fn t2fn2]

a Melting temperature (*T*_m_) and enthalpy of melting (Δ*H*_m_), crystallization temperature (*T*_c_), and enthalpy of crystallization (Δ*H*_c_) were obtained from DSC during the first heating, and cooling scans, respectively. The onset degradation temperature (*T*_onset_), degradation temperature (*T*_deg_), mass loss at *T*_deg_, and residual mass at 700 °C were obtained from TGA.

b-d Different letters in the same column indicate a significant difference among the samples (*p* < 0.05).

Concerning the electrospun biopapers having resveratrol, DES, and DES-solubilized resveratrol, all samples exhibited similar *T*_m_ and *T*_c_ values (approximately 171 and 118 °C, respectively). However, the presence of additives significantly affected both Δ*H*_m_ and Δ*H*_c_ values, causing a decrease of approximately 25 J/g for all samples. The enthalpies of melting and crystallization are directly related to polymer crystallinity; the higher the enthalpy, the higher the energy required for the melting or forming of the crystals, respectively.^16,37^ Hence, this result indicates that the presence of additives inhibited the chain-folding process of PHBV molecules, hindering the molecular arrangement during the formation of crystals, and thus, resulting in ill-defined or imperfect crystals that require less energy to melt or crystallize. To our knowledge, there is no existing data in the literature regarding DES- or resveratrol-containing PHAs. However, resveratrol has previously been incorporated into extruded films of poly­(l-lactide), which is also a biodegradable thermoplastic polyester. In this study, Soto-Valdez et al.^38^ reported that the addition of 1 and 3 wt % of resveratrol decreased both *T*_m_ (by approximately 1 °C) and crystallinity of PLA films, with a more pronounced effect at the higher antioxidant loading of 3 wt %.

Thermogravimetric analysis (TGA) was conducted to assess the thermal stability of the electrospun biopaper samples. The corresponding TGA curves and their derivatives are displayed in Figure 2c,d, respectively. Table 2 summarizes the thermal parameters of the samples, including onset degradation temperature (*T*_onset_), degradation temperature (*T*_deg_), the percentage of mass loss at degradation temperature, and residual mass at 500 °C. In Figure 2c, all electrospun samples exhibited a single step degradation primarily due to random chain scission by β-elimination.^39^ The electrospun pure PHBV biopapers showed a *T*_onset_ of 273 °C. Thermal degradation temperature occurred around 286 °C, resulting in a mass loss of approximately 66%, with a residual mass at 700 °C amounting to only 1.2%. These values closely align with those reported in previous studies, indicating the thermal decomposition of PHBV (ENMAT Y1000P) in a single step, with *T*_onset_ and *T*_deg_ values ranging between 260 and 270 °C, and 277–296 °C, respectively.^17,25,40^ However, as shown in Figure 2c,2d and Table 2, the thermal stability of PHBV biopapers was altered by the incorporation of additives. The addition of 1% of resveratrol resulted in a decrease in *T*_onset_ and *T*_deg_ values by approximately 18 and 16 °C, respectively. This decrease in thermal stability for PHBV+Res biopaper samples was contrary to expectations, considering the reported thermal degradation temperature of pure resveratrol was higher than 280 °C.^7,41^ In this context, Agustin-Salazar et al. prepared PLA films containing 1 and 3 wt % resveratrol^42^ and observed a noticeable decrease in the degradation temperatures for both resveratrol loadings. This change was attributed to phenol hydroxy-initiated transesterification, arising from the stronger nucleophilic character of the aromatic hydroxyl groups in resveratrol compared to the aliphatic terminal groups of PLA. A similar mechanism might explain the thermal stability results observed here.

Regarding the effect of DES, ChCl:EG-containing PHBV biopapers exhibited significantly lower thermal stability than pure PHBV biopapers. This reduction in thermal parameters can be attributed to the lower thermal stability of the DES itself. For instance, Delgado-Mellado et al. and Abbas et al. both reported that the thermal degradation of ChCl/EG (1:2) starts at approximately 100 °C.^43,44^ Despite this, one can also see that ChCl/EG is employed for extended durations at elevated temperatures, reaching as high as 180 °C.^45,46^ Nevertheless, considering the brief annealing duration of only 10 s, it might be suggested that resveratrol and ChCl/EG-solubilized resveratrol remained stable at the selected annealing temperatures (155 °C). Therefore, it is possible that thermal degradation during the postprocessing step was minimized, which is particularly relevant for the purpose of this study.

Although the incorporation of ChCl:EG resulted in a noticeable reduction in the thermal stability of PHBV biopapers, this decrease is not expected to significantly hinder their performance in real-world applications. In most food packaging scenarios, materials are used and stored under ambient or refrigerated conditions, where temperatures remain well below the degradation thresholds observed here. Moreover, the thermal stability of the DES-containing biopapers is still sufficient to withstand sterilization treatments at 120 °C,^47^ a common requirement in food packaging processes.

### 3.4 WAXD

Conventional WAXD experiments were performed to evaluate the possible changes in the crystalline structures of the electrospun PHBV-based biopapers due to the presence of resveratrol, DES, and DES-solubilized resveratrol. Figure 3 shows the WAXD diffractograms within the 2θ range of 5°–33°. As shown in the figure, all samples exhibited a PHB-like crystalline structure with an orthorhombic crystalline lattice, having a space group of *P*2_1_2_1_2_1_ (D_4_^2^).^25,48^ Furthermore, the most characteristic diffractions of PHB, specifically (020) and (110) planes, were identified at around 13.6° and 17° 2θ values. The sharp peak at around 27° was attributed to boron nitride (BN), a nucleating agent known to be present in the commercial PHBV (ENMAT Y1000P).^48^

**Figure 3:**
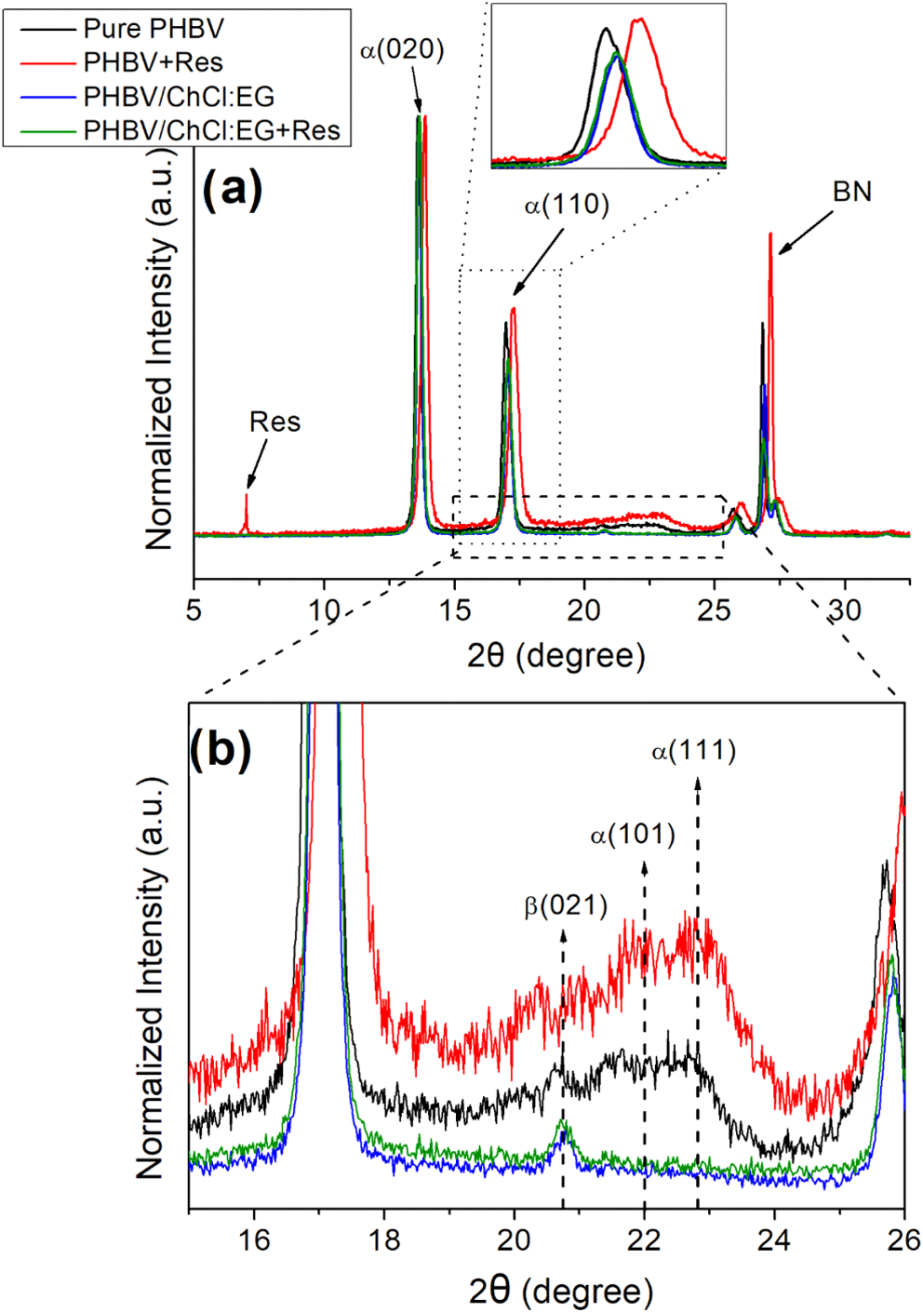
Wide-angle X-ray diffraction (WAXS) patterns of electrospun biopaper samples: pure PHBV, PHBV + Res, PHBV/ChCl:EG, PHBV/ChCl:EG + Res. (a) Full diffraction patterns showing major crystalline peaks; (b) zoomed-in view (15°–26° 2θ) highlighting differences in crystalline structures.

Figure 3a illustrates that the additives significantly altered the crystallinity of the biopapers. Specifically, for PHBV+Res biopapers, the incorporation of nonsolubilized resveratrol led to discernible peak shift toward higher angles, as depicted in the inset of Figure 3a. This indicates that the addition of resveratrol altered the crystalline structure of PHBV and also decreased the distance between atomic layers in the crystal, namely the *d*-spacing (Table 3).^49^ Another interesting observation is that the overall crystallinity, as well as the crystallite sizes on the (020) and (110) planes, was lower for PHBV + Res biopapers compared to the neat ones. This could be due to the presence of resveratrol disrupting the regular packing of PHBV chains, making it harder for them to arrange into large, well-ordered crystalline regions due to the confinement effects of the resveratrol.^50^ More importantly in Figure 3, PHBV + Res biopapers displayed a reflection at 7°. This peak is associated with the resveratrol,^7^ suggesting that the added resveratrol remained crystalline inside PHBV matrix.

**Table 3:** Unit Cell Parameters *a*, *b*, and *c*, Crystallinity, and Interplanar Distances for Electrospun Biopaper Samples: Pure PHBV, PHBV + Res, PHBV/ChCl:EG, PHBV/ChCl:EG + Res

	PHB-like lattice (Å)		interplanar distance (Å)		crystallite size (Å)		
electrospun biopaper sample	*a*	*b*	d020	d110	D020	D110	crystallinity(%)
pure PHBV	5.69	13.01	6.51	5.21	347	244	67
PHBV + Res	5.60	12.79	6.39	5.13	290	209	62
PHBV/ChCl:EG	5.67	12.98	6.49	5.19	385	283	76
PHBV/ChCl:EG + Res	5.67	12.97	6.48	5.19	322	252	78

Similar to resveratrol, the addition of DES also caused significant changes in the crystallinity of PHBV. As shown in Figure 3, both PHBV/ChCl:EG and PHBV/ChCl:EG + Res biopapers displayed a slight peak shift toward higher angles. Contrary to the observations for the addition of pure resveratrol, the addition of DES provoked a small increase in overall crystallinity, by approximately 10% for each cases, where the crystallite sizes were also increased. This could be due to DES could facility by lubrication the aligning of the PHBV chains to pack more closely and orderly, leading to an increase in overall crystallinity. Another surprising effect of DES can be seen in the range of 15–26° 2θ values (Figure 3b). The reflection peaks for the (101) and (111) planes disappeared in both PHBV/ChCl:EG and PHBV/ChCl:EG + Res samples. These observations suggest an interaction between DES and PHBV, perhaps DES could modify the nucleation process, crystallization kinetics and hence the order phase structure of PHBV. Nonetheless, the most pertinent observation for this study’s objectives is that when resveratrol is solubilized in DES, PHBV/ChCl:EG + Res biopapers did not exhibit the crystalline peak of resveratrol that was present in PHBV + Res biopapers at 7° (see Figure 3a). This absence of crystalline peaks suggests that resveratrol was successfully solubilized in the DES and remained as such within the biopapers.

### 3.5 Mechanical Properties

The mechanical characteristics of the developed PHBV-based biopapers are summarized in Table 4, focusing on the elastic modulus (*E*), tensile strength at break (σ_b_), elongation at break (ε_b_), and toughness. The pure PHBV biopapers exhibited an elastic modulus of *ca*. 2.5 GPa, tensile strength at break of *ca*. 18 MPa, elongation at break of 1.9%, and toughness of 0.23 mJ/m^3^. These parameters clearly show that pure PHBV biopapers were rigid and brittle as widely reported in previous literature. In another study, Figueroa-López et al. developed biopaper layers of electrospun PHBV (ENMAT Y1000P) via electrospinning and subsequent thermal-post processing techniques.^51^ The reported mechanical parameters included an *E* of 1.3 GPa, σ_b_ of 19.1 MPa, and ε_b_ of 2.0%. While the σ_b_ and ε_b_ values were almost the same, the here-in reported elastic modulus appeared to be higher. This could be attributed to the differences in biopaper thickness, or to variations in macro- and mesoscale morphologies.^25,52^

**Table 4:** Mechanical Properties in Terms of Elastic Modulus (*E*), Tensile Strength at Break (σ_b_), Eongation at Break (ε_b_), and Toughness (*T*) of the Electrospun Pure PHBV, PHBV + Res, PHBV/ChCl:EG, PHBV/ChCl:EG + Res Biopapers

biopaper	*E* (MPa)	σ_b_ (MPa)	ε_b_ (%)	toughness (mJ/m^3^)
pure PHBV	2512 ± 404[Table-fn t4fn1]	17.6 ± 0.7[Table-fn t4fn1]	1.9 ± 0.2[Table-fn t4fn1]	0.23 ± 0.05[Table-fn t4fn1]
PHBV + Res	3527 ± 572[Table-fn t4fn1]	34.7 ± 3.9[Table-fn t4fn1]	1.7 ± 0.2[Table-fn t4fn1]	0.33 ± 0.10[Table-fn t4fn1] [Table-fn t4fn1]
PHBV/ChCl:EG	3349 ± 406[Table-fn t4fn1]	38.9 ± 7.4[Table-fn t4fn1]	1.9 ± 0.3[Table-fn t4fn1]	0.45 ± 0.06[Table-fn t4fn1]
PHBV/ChCl:EG + Res	2694 ± 497[Table-fn t4fn1]	22.2 ± 0.9[Table-fn t4fn1]	1.6 ± 0.4[Table-fn t4fn1]	0.22 ± 0.05[Table-fn t4fn1]

a-b Different letters in the same column indicate a significant difference among the samples (*p* < 0.05).

When resveratrol, DES, and DES-solubilized resveratrol were added into the PHBV matrix, the measurements for elastic modulus and elongation at break showed no significant differences. However, the tensile strength at break was found to be higher in the PHBV + Res and PHBV/ChCl:EG biopapers. Notably, the PHBV/ChCl:EG + Res biopapers displayed tensile strength values that were comparable to those of the pure PHBV biopapers. The enhanced strength of the PHBV/ChCl:EG biopapers over the pure PHBV biopapers can be attributed to their higher crystallinity, as revealed in the WAXS analysis (refer to Table 3). Generally, a higher crystallinity leads to more rigid materials in terms of tensile properties.^53,54^ Despite the PHBV + Res biopapers having lower crystallinity than the neat films, they demonstrated superior strength. This improvement in mechanical properties may be due to effective stress transfer between PHBV and the resveratrol active filler.^55^ Conversely, while PHBV/ChCl:EG biopapers were stronger than the pure PHBV biopapers, likely due to their higher crystallinity (as shown in Table 3), this correlation did not extend to PHBV/ChCl:EG + Res biopapers, which exhibited mechanical properties similar to those of the neat biopapers.

Additional factors may contribute to the enhanced mechanical strength observed in the developed biopapers. One possible explanation is the enhancement of intermolecular interactions between the additives and the PHBV matrix, which can reinforce the polymer network by influencing the nucleating process and crystallization behavior of PHBV (see section 3.4).^17^ Another contributing factor could be the well-dispersed additive load within the polymer matrix, enabled by the electrospinning technique. Uniform dispersion is known to improve the mechanical strength of polymeric composites.^56,57^ The occupation of free volume within the polymer matrix by small amounts of additives promotes tighter chain packing and potentially increases crystallinity, both of which can contribute to improved mechanical strength.^58^ For instance, Venezia et al. reported improved mechanical strength in PHBV-based biopapers upon incorporation of TiO_2_/humic substance nanoparticles. The authors attributed this enhancement to interactions between the filler and biopolymer, good load dispersion, increased polymer crystallinity, and strong interfacial adhesion between the nanoparticles and the biopolymer matrix.^57^

### 3.6 Optical Properties

Visual aspects of the developed electrospun biopapers are presented in Figure 4. All electrospun biopapers displayed similar visual properties, with no detectable color change, indicating the absence of thermal degradation.^17^ To quantitatively assess the optical properties of these biopapers, contact transparency (*T*) measurements were performed, with the findings compiled in Figure 4. The electrospun biopaper produced solely from PHBV exhibited a transparency value of 19.4%, which is similar to previously reported values for electrospun PHBV films (ENMAT Y1000P).^27^ In biopapers containing resveratrol, DES, and DES-solubilized resveratrol, the contact transparency values were slightly lower compared to pure PHBV biopapers, though the reductions were statistically insignificant. Similar trends have been reported in previous studies involving PHBV films with additives, where a slight decrease in transparency is generally attributed to the presence of additives affecting the refractive index.^18,27^ Similarly, the here-observed reduction in transparency for PHBV + Res, PHBV/ChCl:EG, and PHBV/ChCl:EG + Res biopapers can be attributed to light scattering caused by the additives.^18^ From a food preservation standpoint, the reduced transparency of PHBV biopapers containing DES-solubilized or nonsolubilized resveratrol could be advantageous, as these films may provide enhanced light barrier properties, thereby reducing the potential for oxidation of photolabile compounds such as resveratrol.^59^ Additionally, no color change was detected in any of the samples, indicating that the biopapers were not thermally affected during the annealing postprocessing.

**Figure 4:**
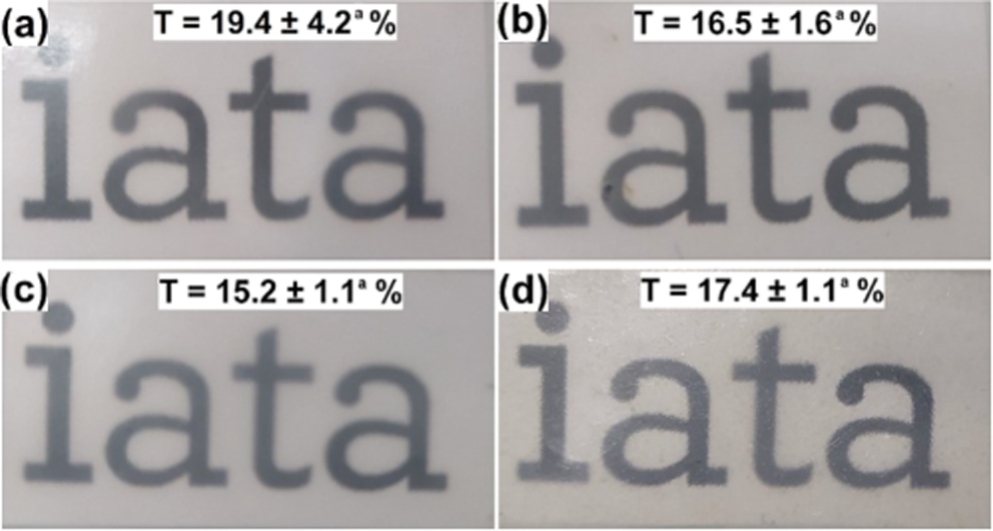
Visual aspect and transparency (*T*(%)) of the electrospun (a) pure PHBV, (b) PHBV/Res, (c) PHBV/ChCl:EG, (d) PHBV/ChCl:EG + Res biopapers. ^a^No significant difference among the samples (*p* > 0.05).

### 3.7 Barrier Properties

Barrier performance is a critical factor in the application of films for food packaging. The barrier properties of developed electrospun biopapers, particularly in terms of their permeability to water vapor (WVP) and oxygen (OP), are summarized in Table 5. It can be seen that electrospun pure PHBV biopapers presented a WVP value of 0.29 × 10^–14^ kg·m·m^–2^·Pa^–1^·s^–1^. When comparing these results with other studies that used the same PHBV material (ENMAT Y1000P) for the electrospun biopapers, one can find a noticeable variation in WVP values, ranging approximately from 4 to 6 × 10^–14^ kg·m·m^–2^·Pa^–1^·s^–1^ ^17,24,51^ . This discrepancy can be attributed to the biopaper manufacturing process, specifically the annealing step. Annealed mats forming continuous biopapers, formed through fiber coalescence, could generate a variable degree of coalescence efficiency depending on specific processing and postprocessing conditions applied.^26,60^

**Table 5:** Permeability of the Annealed Electrospun Biopaper Samples: Pure PHBV, PHBV + Res, PHBV/ChCl:EG, PHBV/ChCl:EG + Res

film	WVP × 10^14^ (kg·m·m^–2^·Pa^–1^·s^–1^)	OP × 10^19^(m^3.^m·m^–2^·Pa^–1^·s^–1^)
pure PHBV	0.29 ± 0.00[Table-fn t5fn1]	2.16 ± 0.01[Table-fn t5fn1]
PHBV + Res	0.23 ± 0.05[Table-fn t5fn1]	>1000
PHBV/ChCl:EG	0.68 ± 0.04[Table-fn t5fn1]	5.93 ± 0.24[Table-fn t5fn1]
PHBV/ChCl:EG + Res	0.59 ± 0.01[Table-fn t5fn1]	6.64 ± 0.05[Table-fn t5fn1]

a-c Different letters in the same column indicate a significant difference among the samples (*p* < 0.05).

When resveratrol was solely incorporated into the PHBV matrix, no significant differences in WVP were observed in the resulting electrospun biopapers. Contrastingly, the inclusion of DES into the PHBV biopapers significantly altered the barrier properties. Specifically, the WVP values of electrospun PHBV/ChCl:EG and PHBV/ChCl:EG + Res biopapers were found to be higher than those of pure PHBV and PHBV + Res samples. Given that water vapor primarily moves through PHAs by diffusion, owing to their low capacity to absorb water,^16^ the increase in permeability to water vapor can be attributed to the hydrophilic nature of the DES used in this study.^61^ Notably, the WVP values of the here-developed electrospun biopapers, even with the inclusion of DES and resveratrol, were maintained within the same order of magnitude as those of their homopolymer PHB, as well as the petroleum-based polyethylene terephthalate (PET) film counterparts, which is one the most widely used synthetic, nondegradable polymer in the packaging industry with similar water vapor permeability, i.e., 0.5 × 10^–14^ and 0.23 × 10^–14^ kg·m·m^–2^·Pa^–1^·s^–1^.^16,60,62^

In terms of barrier performance to oxygen, electrospun pure PHBV biopapers demonstrated an OP value of 2.16 × 10^–19^ m^3.^m.m^–2^.Pa^–1^.s^–1^. This finding is consistent with the results reported by Figueroa-Lopez et al., who observed an OP value of 3.65 × 10^–19^ m^3.^m·m^–2^.Pa^–1^·s^–1^ for electrospun PHBV biopapers (ENMAT Y1000P).^24^ However, when resveratrol was incorporated into the PHBV matrix, the oxygen permeability of the resulting electrospun PHBV+Res biopapers exceeded the measurable range, with OP values surpassing 1000 × 10^–19^ m^3.^m·m^–2^·Pa^–1^·s^–1^. Oxygen, being a small, noncondensable permeant, is particularly sensitive to free volume, morphological and phase structure differences, and defects within a material.^16,26^ Hence, the very high oxygen permeability in PHBV+Res biopapers can be attributed to the introduction of heterogeneities within the biopaper matrix by solid resveratrol, which likely created preferential pathways for oxygen, potentially on the PHBV-resveratrol interface. In any case, although the oxygen permeability of PHBV + Res biopapers is high, this limitation may be mitigated in multilayered food packaging systems, where additional barrier layers can compensate for reduced gas barrier performance.^51^

Contrary to the results with resveratrol used as a filler, the incorporation of deep eutectic solvents (DES) and DES-solubilized resveratrol into PHBV led to reduced permeability but this remained within the scale of the unfilled material. This observation suggests that the DES containing biopaper was not so detrimental in blocking oxygen. In fact, the WAXS analysis revealed that the effect of DES, which causes the PHBV chains to pack closely and orderly, leading to higher crystallinity, could play a role in the observed barrier performance. For instance, da Costa et al. reported a reduction in the oxygen permeability of compression-molded PHBV films upon incorporation of oregano essential oil, which remains in a liquid state within the films, similar to DES.^63^ This effect was attributed to the increased crystallinity induced by the oil, with the resulting crystalline domains acting as barriers that hinder the diffusion of oxygen. Overall, the developed electrospun PHBV/ChCl:EG and PHBV/ChCl:EG + Res biopapers demonstrated oxygen permeability values that are competitive with those of petroleum-based poly­(vinyl alcohol) (PVOH), known for its excellent oxygen barrier properties.^62^ This suggests that DES and DES-solubilized resveratrol could be also effective additives for passive oxygen barrier biodegradable PHBV biopapers, offering a sustainable alternative to traditional petroleum-based packaging materials.

### 3.8 Antioxidant Activity

The antioxidant performance of the developed PHBV-based biopapers was examined by the DPPH assay. Figure 5 illustrates the antioxidant activity of the electrospun biopapers, as a function of biopaper, and resveratrol concentration (depicted on lower, and upper *X*-axes, respectively) over a 24-h period in contact with a DPPH solution. It can be seen from the figure that both pure PHBV and PHBV/ChCl:EG biopapers presented a certain level of antioxidant activities, ranging from approximately 12% at the lowest concentration to 20% at the highest concentrations. As shown in Figure 5 pure PHBV and PHBV/ChCl:EG biopapers presented a certain level of antioxidant activity that was approximately 12% and 20% at the lowest and highest concentrations, respectively. However, it is known that PHBV alone offers negligible antioxidant capacity.^64^ For the case of the antioxidant activity of ChCl:EG, prior studies have indicated that deep eutectic solvents composed of choline chloride and 1,2-propanediol, as well as those formulated with choline chloride and glycerol, do not possess any antioxidant activities.^65,66^ Hence, given the similar chemical structure of ethylene glycol, the DES, ChCl:EG, studied here is not expected to exhibit antioxidant activity, as evidenced from Figure 5. Nevertheless, the low antioxidant response of both pure PHBV and PHBV/ChCl:EG biopapers might be attributed to the biopapers absorbing DPPH during exposure, as suggested by other authors.^2,28^

**Figure 5:**
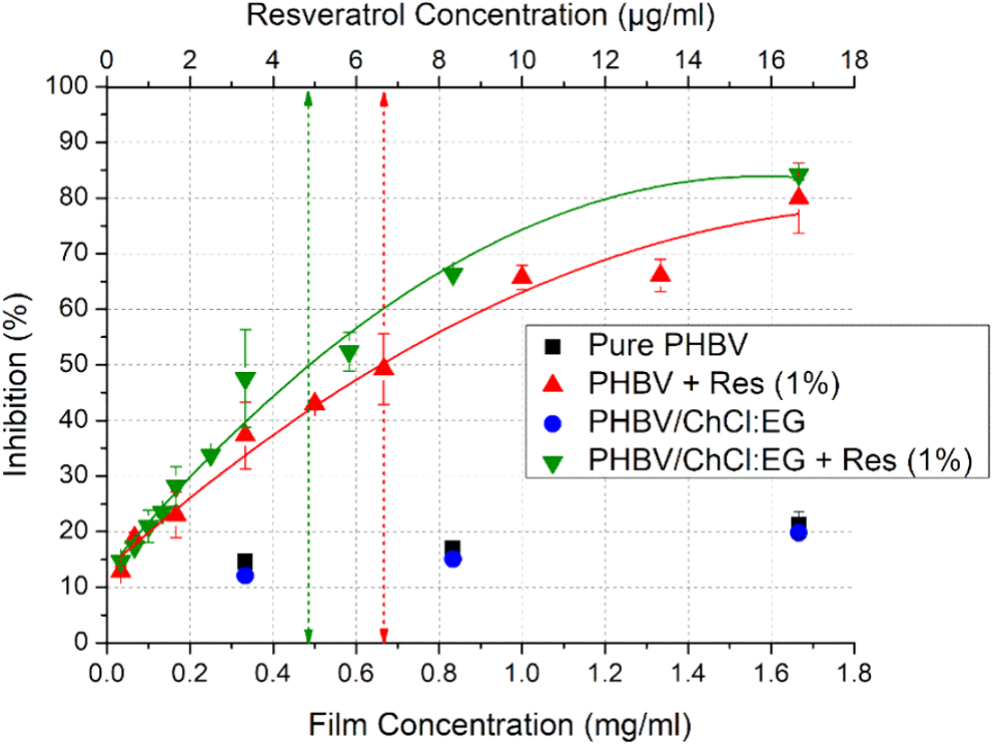
Radical scavenging activities of the electrospun biopapers of pure PHBV, PHBV/ChCl:EG, PHBV + Res, and PHBV/ChCl:EG + Res as a function of increasing biopaper (lower *X*-axis, mg/mL of DPPH solution) or resveratrol (upper *X*-axis μg/mL of DPPH solution) concentrations. Arrows indicate the determined IC50 values for the electrospun PHBV + Res (*R* ^2^: 0.9773) and PHBV/ChCl:EG + Res (*R* ^2^: 0.9783) biopapers.

When resveratrol was incorporated into PHBV biopaper at the concentration of 1 wt %, a significant antioxidant activity was detected. As expected, higher radical inhibition percentages were observed with the increasing concentrations of biopapers, and thus the resveratrol. The arrows in the Figure 5 indicate the IC50 values of the PHBV + Res and PHBV/ChCl:EG + Res biopaper samples. This value represents the amount of the active ingredient required to scavenge the 50% of the initial DPPH concentration.^29^ Lower the IC50, higher the antioxidant capacity. The IC50 value for PHBV + Res biopapers was 0.68, whereas this was 0.48 for the PHBV/ChCl:EG + Res biopapers. This suggests that solubilizing resveratrol in ChCl:EG enhances material efficiency by 30%, attaining the same scavenging performance. Moreover, this efficiency improvement appears to be proportional to the level of desired inhibition, for example, at IC60 or IC70, indicating a trend of increased efficiency with higher inhibition targets. This can be attributed to the exceptional solubility of resveratrol in ChCl:EG, which leads to better diffusion through the polymer matrix, thus contributing to improved antioxidant activity.^29,67^ For instance, Zainal-Abidin et al. reported an excellent solubility of the polyphenol compounds in ChCl/EG, demonstrating also a significant increase in antioxidant performance when the polyphenols from were extracted using ChCl/EG (88% DPPH inhibition) compared to those extracted with a nonsolubilizing medium (methanol, 30% DPPH inhibition).^68^ The authors then suggested that increased solubility of antioxidant compounds enhances their antioxidant activity. In another example, Celebioglu et al. improved the water solubility of resveratrol through hydroxypropylated cyclodextrin (HPβCD) complexation.^29^ According to their results, this led to increased diffusion through the polymer matrix and exhibited enhanced antioxidant activity.

In addition to enhanced solubility and diffusion, the deep eutectic solvent (ChCl/EG) also played a protective role during processing. Although the PHBV/ChCl:EG + Res biopapers were annealed at 155 °C for 10 s to achieve a nonporous structure, this short exposure was insufficient to cause significant degradation of resveratrol. For context, resveratrol has been reported to degrade by 39% only after 20 min at 150 °C, indicating that degradation is strongly time-dependent.^69^ Moreover, DES have been shown to stabilize antioxidants through extensive hydrogen bonding interactions under thermal conditions.^70^ In our case, resveratrol was both solubilized in ChCl:EG and physically entrapped within the PHBV matrix, which likely provided similar stabilization. Together, these factors contributed to the preservation—and observed enhancement—of antioxidant activity in the final biopapers.

## 4 Conclusions

This study reports the remarkable potential of deep eutectic solvents (DES) in enhancing the solubility and antioxidant activity of a natural antioxidant, resveratrol. Notably, a specific DES formulation, composed of ChCl and EG, enabled an unreported resveratrol solubility of 400 mg/mL—setting a benchmark in antioxidant applications. Leveraging on this, electrospun biopapers were developed using the biodegradable and biobased polymer PHBV, incorporating DES-solubilized resveratrol (1 wt %) for innovative, sustainable packaging solutions. The resulting biopapers were continuous and self-supporting. The incorporation of DES reduced the thermal stability of PHBV, though this was not a major concern due to the mitigating effects of the processing technique used. All developed biopapers exhibited favorable optical, water vapor barrier, and mechanical properties, with a noteworthy difference in oxygen permeability (OP). For instance, biopapers containing nonsolubilized resveratrol displayed unmeasurable OP values exceeding 1000 × 10^–19^ m^3^·m·m^–2^·Pa^–1^·s^–1^, while DES-solubilized resveratrol produced OP values similar to pure PHBV biopapers. Antioxidant testing further revealed that DES-solubilized resveratrol provided superior antioxidant activity, achieving 30% greater material efficiency compared to nonsolubilized resveratrol with the same 1 wt % loading. These findings highlight the potential of the here-developed materials for extending food shelf life while reducing costs—key attribute for modern active packaging solutions. Overall, this research demonstrates the successful solubilization of poorly soluble antioxidants like resveratrol using DES, presenting a sustainable and environmentally friendly alternative to traditional organic solvents. By incorporating DES within eco-friendly biopolyesters like PHBV, we further enhance the prospects of these electrospun biopapers for active, sustainable packaging applications. Future research could explore the scalability and time-dependent antioxidant performance of this approach, as well as its potential applicability to other poorly soluble compounds, broadening the scope of DES in material science and sustainable packaging innovations.

## Supplementary Material



## Notes

Visual observation of resveratrol solubility in the selected deep eutectic solvent (DES), ChCl/EG, is illustrated through vial images, additional experimental details and characterizations include fiber diameter distribution and Attenuated Total Reflectance Fourier Transform Infrared (ATR-FTIR) spectroscopy (PDF)
