# Supplementary material for: Antioxidant Electrospun Poly(3-hydroxybutyrate-co-3-hydroxyvalerate) (PHBV) Films Loaded with Resveratrol Solubilized in Deep Eutectic Solvents
Source: ACS Appl Polym Mater. 2025 Aug 7;7(16):10416–29. doi: 10.1021/acsapm.5c01101 (PMC12379765; doi:10.1021/acsapm.5c01101)
Supplement: Supplementary file 1 [file ap5c01101_si_001.pdf]

Supporting Information

Antioxidant Electrospun Poly(3-hydroxybutyrate-co-3-hydroxyvalerate) (PHBV) Films Loaded with Resveratrol Solubilized in Deep Eutectic Solvents

Ahmet O. Basar[1], Cristina Prieto[1], Evangelia Bardakou[1], Luis Cabedo[2], Jose M. Lagaron[1*]*

[1]Novel Materials and Nanotechnology group, Institute of Agrochemistry and Food Technology (IATA), Spanish Council for Scientific Research (CSIC), Calle Catedrático Agustín Escardino Benlloch 7, 46980 Paterna, Spain.

[2] Polymers and Advanced Materials Group (PIMA), School of Technology and Experimental Sciences, Universitat Jaume I (UJI), Avenida de Vicent Sos Baynat s/n, 12071 Castellón, Spain.

E-mail: lagaron@iata.csic.es and cprieto@iata.csic.es

EXPERIMENTAL SECTION

Scanning Electron Microscopy (SEM)

Fiber diameter distribution was determined by measuring 100 fibers per sample using ImageJ software (NIH, Bethesda, MD, USA), based on SEM images captured at their original magnification.

ATR-FTIR Spectroscopy

Attenuated total reflectance Fourier-transform infrared spectroscopy (ATR-FTIR) analysis were performed using a Bruker FTIR Tensor 37 equipment (Rheinstetten, Germany). The samples were deposited onto the diamond crystal, ensuring effective contact through the utilization of the ATR sampling Accessory low-temperature Golden Gate (Specac Ltd., Orpington, UK). The spectra acquired across the wavenumber range of 4000-600 cm^{-1}, averaging 10 scans at a resolution of 2 cm^{-1}. The measurements were conducted in triplicate, and analysis of the spectral data was performed using the OPUS 4.0 data collection software (Bruker, Ettlingen, Germany).

EXPERIMENTAL RESULTS

Visual Observation

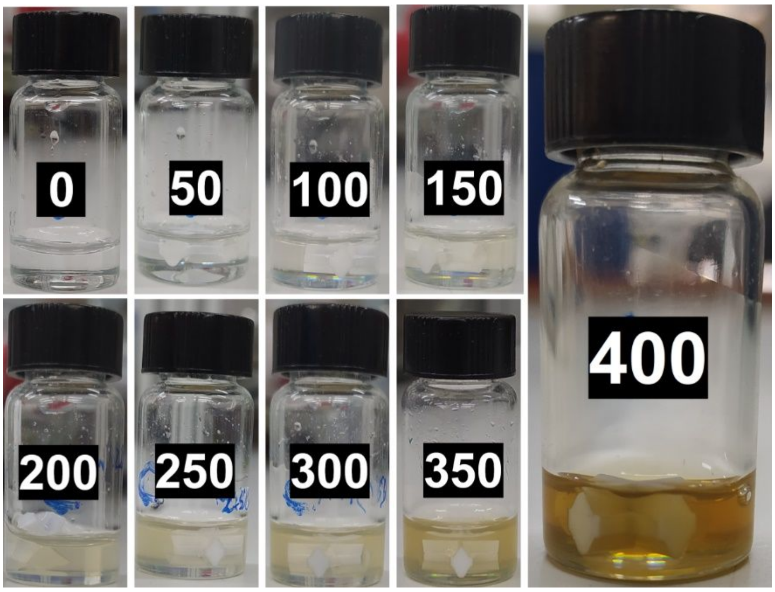

Figure S1. Visual observation of resveratrol solubility in a selected deep eutectic solvent, ChCl:EG. Each vial is labeled with the corresponding resveratrol concentration in mg/ml. The 400 mg/ml solution was aged for at least 2 months, while the others were freshly prepared.

SEM

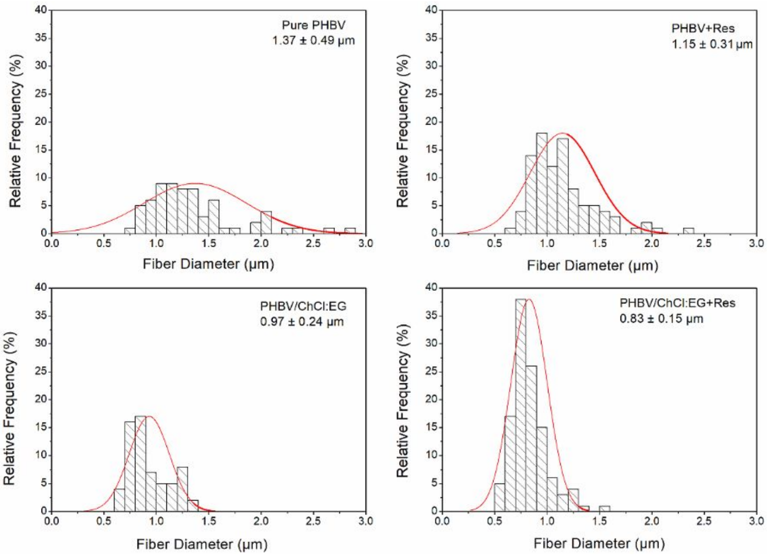

Figure S2. Fiber size distribution of electrospun biopaper samples: pure PHBV, PHBV+Res, PHBV/ChCl:EG, PHBV/ChCl:EG+Res.

ATR-FTIR Analysis

ATR-FTIR spectroscopy was conducted on the developed PHBV-based biopapers, as well as the raw materials, resveratrol and ChCl:EG, and Figure S3 displays the corresponding spectra. The pristine DES, ChCl:EG, revealed the relevant characteristics peaks at 3300 cm^{-1} and 1478 cm^{-1}, corresponding to O-H stretching and CH_2 bending of an alkyl group, respectively. Additionally, bands at 1084 cm^{-1} and 1036 cm^{-1} correspond to functional groups stretching as C-O, and asymmetric C-C-O, respectively[1]. In the case of pure resveratrol, a broad feature of O-H stretching was observed at 3190 cm^{-1}, and distinctive vibrational bands were revealed in the 1650-1500 cm^{-1} region, corresponding to the C-C aromatic double bond

stretching[2,3]. Regarding PHBV biopaper samples, the characteristic ester group C=O band peaks were observed with the highest intensity at 1720 cm^{-1} for each sample[4]. Additionally, each biopaper sample exhibited consistent features in the fingerprint region (1400-700 cm^{-1}), including C-H bending (1378 cm^{-1}), C-O stretching (1274, 1042 cm^{-1})[5]. However, no characteristic peaks of resveratrol or DES were detected in the ATR-FTIR spectra of PHBV+Res, PHBV/ChCl:EG, and PHBV/ChCl:EG+Res biopaper samples. This is likely due to the low concentrations of resveratrol (1 wt. %) and DES (2.6 wt.%) within the PHBV matrix, whose contributions would be masked by the intense peaks of PHBV.

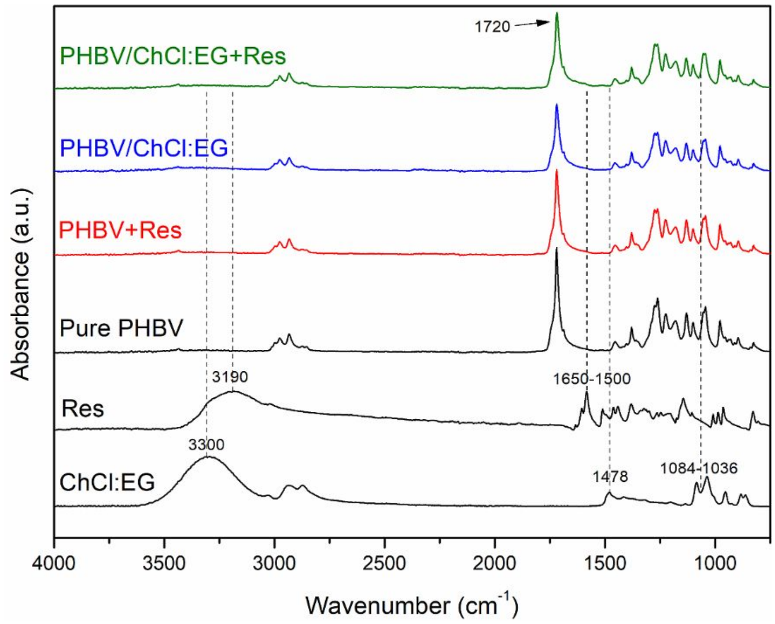

Figure S3. ATR-FTIR spectra of electrospun biopaper samples: pure PHBV, PHBV+Res, PHBV/ChCl:EG, PHBV/ChCl:EG+Res, and pure components of ChCl:EG and resveratrol.

REFERENCES

(1) Delgado-Mellado, N.; Larriba, M.; Navarro, P.; Rigual, V.; Ayuso, M.; García, J.; Rodríguez, F. Thermal Stability of Choline Chloride Deep Eutectic Solvents by TGA/FTIR-ATR Analysis. *J. Mol. Liq.* **2018**, *260*, 37–43. https://doi.org/10.1016/j.molliq.2018.03.076.

(2) Busolo, M. A.; Lagaron, J. M. Antioxidant Polyethylene Films Based on a Resveratrol Containing Clay of Interest in Food Packaging Applications. *Food Packag. Shelf Life* **2015**, *6*, 30–41. https://doi.org/10.1016/j.fpsl.2015.08.004.

(3) Silva, P. M.; Neto, M. D.; Cerqueira, M. A.; Rodriguez, I.; Bourbon, A. I.; Azevedo, A. G.; Pastrana, L. M.; Coimbra, M. A.; Vicente, A. A.; Gonçalves, C. Resveratrol-Loaded Octenyl Succinic Anhydride Modified Starch Emulsions and Hydroxypropyl Methylcellulose (HPMC) Microparticles: Cytotoxicity and Antioxidant Bioactivity Assessment after in Vitro Digestion. *Int. J. Biol. Macromol.* **2024**, *259* (P2), 129288. https://doi.org/10.1016/j.ijbiomac.2024.129288.

(4) Melendez-Rodriguez, B.; Reis, M. A. M.; Carvalheira, M.; Sammon, C.; Cabedo, L.; Torres-Giner, S.; Lagaron, J. M. Development and Characterization of Electrospun Biopapers of Poly(3-Hydroxybutyrate- Co-3-Hydroxyvalerate) Derived from Cheese Whey with Varying 3-Hydroxyvalerate Contents. *Biomacromolecules* **2021**, *22* (7), 2935–2953. https://doi.org/10.1021/acs.biomac.1c00353.

(5) Cherpinski, A.; Torres-Giner, S.; Cabedo, L.; Lagaron, J. M. Post-Processing Optimization of Electrospun Submicron Poly(3-Hydroxybutyrate) Fibers to Obtain Continuous Films of Interest in Food Packaging Applications. *Food Addit. Contam. - Part A Chem. Anal.*

Control. Expo. Risk Assess. **2017**, *34* (10), 1817–1830. https://doi.org/10.1080/19440049.2017.1355115.
